# Symptomatic and functional recovery in depression in later life

**DOI:** 10.1007/s00127-018-1540-z

**Published:** 2018-06-19

**Authors:** Rose M. Collard, Sanne Wassink-Vossen, Aart H. Schene, Paul Naarding, Peter Verhaak, Richard C. Oude Voshaar, Hannie C. Comijs

**Affiliations:** 1 Department of Psychiatry, Radboud University Medical Center, Nijmegen, The Netherlands; 2 Department of Old-age Psychiatry, GGNet, Apeldoorn, Zutphen, The Netherlands; 3 Department General Practice, University Medical Center Groningen, University of Groningen, Groningen, The Netherlands; 4 Netherlands Institute for Health Services Research, NIVEL, Utrecht, The Netherlands; 5 University Medical Center Groningen, University Center for Psychiatry, University of Groningen, Groningen, The Netherlands; 6 GGZinGeest, Amsterdam, The Netherlands; 7 Department Psychiatry/Amsterdam Public Health Research Institute, VU University Medical Center, Amsterdam, The Netherlands

**Keywords:** Late-life depression, Functional limitations, Remission, Netherlands Study of Depression in Older persons

## Abstract

**Objectives:**

Functional limitations give an indication of the total impact of diseases, such as depression, on individuals health and recovery. This study examines the change in several domains of functioning over 2 years in older persons depressed at baseline (non-remitted group and remitted group after 2 years) and in a non-depressed comparison group.

**Methods:**

Data were used from a cohort study (Netherlands Study of Depression in Older persons [NESDO]) consisting of depressed older persons ≥ 60 years (*N* = 378) and a non-depressed comparison group (*N* = 132) with 2 years of follow-up (attrition rate 24%). Functional limitations (outcome) were assessed with the World Health Organization Disability Assessment Schedule 2.0 (WHODAS 2.0) questionnaire every 6 months. Total scores and domain scores were used. Depression was classified according to the Diagnostic and Statistical Manual of Mental Disorders, Fourth Edition (DSM-IV) criteria at baseline and at 2-year follow-up. Severity of depression (predictor) was assessed with the Inventory of Depressive Symptomatology (IDS) at 6-month intervals.

**Results:**

Linear mixed models showed that the level of functional limitations differed between the three groups during 2 years of follow-up. The non-remitted group had the highest level of functional limitations during 2 years, followed by the remitted group. Stable low levels of functional limitations were found for the non-depressed group. Remission from depression was accompanied by improvements in functioning, however, compared to the non-depressed comparison group significant functional limitations remained. Higher severity of depression appeared as risk factor for a declining course of functioning, especially the social aspects of functioning.

**Methodological considerations:**

Participants that were more severely depressed and more functionally impaired at baseline had higher attrition rates than the participants that were included in the analytical sample.

**Conclusion:**

This study showed that depression in later life has long-term debilitating effects on functioning, enduring even after remission from depression. This implies that depression treatment in later life should aim broader than just symptomatic recovery, but also include functional recovery.

## Introduction

Depression is the most common psychiatric disease worldwide [1]. Severity of depression ranges from some clinically relevant depressive symptoms to minor depression and eventually, major depression. With demographic balance shifting towards an older population, the number of older adults with a lifetime history of depression will increase significantly over the next decades [2].

Both depression and increasing age are associated with functional decline [3–7]. The concept of functioning pertains to how people function in everyday life, in the performance of activities, and in the areas of life in which they participate [8]. Measures of functional decline can provide insight in the total impact of diseases, such as depression, on individuals. When older persons themselves are asked about what matters about their health, they state that longevity is certainly important, but more important is what they can or cannot do in daily life [8]. Studies in old-age psychiatry assemble with this development by increasingly recognizing functional decline as an important outcome measure in studies on depression [9, 10].

Empirical evidence already showed that increasing severity of depressive symptoms predicts functional decline [11–13] and that physical disability improves with treatment for depression [14]. However, to date, studies are limited to younger adults or to depressive symptoms count. Taken together, only one study on the longitudinal association between late-life depressive disorder and functional decline was found. Riddle et al. showed that clinical remission was not associated with less cognitive decline [15].

This study aims to examine change in functional status over a period of 2 years in clinically depressed older persons and a non-depressed comparison group at baseline. Within the depressed group, we further examined whether changes in the severity of depression were associated with changes in several domains of functioning over a period of 2 years. We hypothesized that patients who reached clinical remission would report better functioning than those who did not, but worse than the non-depressed comparison group.

## Method

Study participants came from the Netherlands Study of Depression in Older persons (NESDO) [16] an ongoing cohort study aimed at examining the long-term course and consequences of depressive disorders in persons aged 60 years and older. The NESDO study included 378 depressed subjects (age range 60–93 years) from mental health institutes (both in- and outpatients) and from primary care who, at baseline, suffered from a current DSM-IV classification of major depressive disorder (95%), minor depression (5.6%), or dysthymia (26.5%), of which 26.5% have two types of depressive disorders, and a non-depressed comparison group (no lifetime depression) (*N* = 132). Exclusion criteria were a (suspected) primary diagnosis of dementia, or another serious psychiatric disorder, since the course of depression in these persons will be largely determined by the primary disorder. A Mini–Mental State Examination score (MMSE) under 18 and insufficient mastery of the Dutch language were also exclusion criteria. All participants were competent to consent to participation and gave written informed consent. The ethical review boards of the participating institutes approved of this study.

In the comparison group, five persons became depressed during the 2-year follow-up period and were excluded from the current analytical sample.

Data collection consisted of an examination at one of the participating clinics or at the homes of the participants, including a structured diagnostic interview, physical tests (such as blood pressure and gait speed), and paper and pencil questionnaires. These assessments were conducted at baseline and at 2 years follow-up (overall attrition rate of 21.4%) [17]. Furthermore, the course of depressive symptoms and functional limitations were followed up every 6 months by means of a postal questionnaire.

The present study was performed on all participants from the depressed group (*n* = 285) and the comparison group (*n* = 111) who had data at 2 years follow-up available. Persons that were lost to follow-up had lower cognitive functioning (MMSE score 27.2 versus 28.0, *p* = 0.006), more functional limitations (WHODAS standardized score: 29.7 versus 24.3, *p* = 0.006), lower educational levels (10.3 versus 11.1 years, *p* = 0.024), and were more severely depressed (Inventory of Depressive Symptoms self-rating [IDS-SR] score: 27.5 versus 23.1). We found no baseline differences between both groups with regard to age, sex and presence of multimorbidity.

## Measures

### Depression

The Composite International Diagnostic Interview (CIDI), version 2.1 was used to assess the presence of a depressive disorder at baseline and at 2 years follow-up [18]. The CIDI is a structured interview that assesses mental disorders in adults according to the criteria of Diagnostic and Statistical Manual of Mental Disorders, Fourth Edition (DSM-IV). The CIDI has good validity and reliability for depressive disorders [19, 20]. To determine a DSM-IV diagnosis of current minor depression, questions about the presence of the symptoms in the last month were added to the CIDI, as in the Netherlands Study of Depression and Anxiety (NESDA) [21]. Remission was defined as absence of depression diagnosis (major depression, minor depression and dysthymia) at 2 years follow-up.

Severity of depression was measured every 6 months by the validated 30-item IDS-SR [22]. In the IDS-SR, items are scored on a four-point scale, with each item equally weighted and summed up to a total score. A higher total score indicates more severe depression.

### Functional status

Functional status was measured every 6 months using the self-report World Health Organization Disability Assessment Schedule 2.0 (WHODAS II) [23–25]. The WHODAS 2.0 is a commonly used and interdisciplinary instrument that contains 36 items, covering six domains of functioning during the last 30 days: (1) cognition (understanding and communicating); (2) mobility (moving and getting around); (3) hygiene, dressing, eating and staying alone (self-care); (4) interpersonal actions (getting along with people); (5) work, leisure, domestic responsibilities (household activities); (6) joining in community activities (participation in society).

The items about work were omitted for the NESDO study, because most of the participants were retired from work. Total score and separate domain scores were calculated. Overall standardized scores ranged from 0 to 100, where higher scores reflect greater disability (http://www.who.int/classifications/icf/whodasii/en/).

### Covariates

Demographic data (age, sex, and educational level) were collected during the baseline assessment. Global cognitive functioning was assessed by the MMSE (range 0–30) [26] with higher scores indicating better cognitive functioning. Interrater reliability and test–retest reliability of the MMSE are good [27, 28].

Multimorbidity was assessed using a self-report questionnaire about the presence of somatic diseases [lung disease, cardiovascular disease, diabetes, arthritis, rheumatism, cancer, ulcer, intestinal disorder, liver disease, epilepsy, allergy, thyroid gland disease and (head) injury], as originally developed by Statistics Netherlands (Centraal Bureau voor de Statistiek, http://www.cbs.nl). This questionnaire has high accuracy for chronic somatic disease [29]. Multimorbidity was considered present when > 1 somatic disease under treatment was reported.

### Statistical analyses

Baseline demographics and clinical characteristics of the remitted participants, non-remitted participants and the non-depressed comparison group were compared using one-way ANOVA for continuous variables and *χ*^2^ tests for categorical variables.

To examine whether depression status was associated with the course of functioning, estimated mean scores of functioning over 2 years and time interactions were assessed with linear mixed models for the three groups: non-remitted participants (depressed at baseline and at follow-up), remitted participants (depressed at baseline and remitted at follow-up) and the non-depressed (never depressed) comparison group. All analyses were performed unadjusted, and subsequently adjusted for socio-demographics (age, sex, education), cognitive functioning (MMSE score), multimorbidity and time. Depression status at follow-up (remitted /non-remitted) and all covariates were entered as fixed factors. Subjects were treated as random factors. The interaction effects between depression status and time were added to the fully adjusted model to assess whether the course of functional limitations differed between remitted, non-remitted, and never-depressed participants.

To examine whether the severity of depression predicted functional limitations in the group that was depressed at baseline, linear mixed models were used with the severity of depressive symptoms as a time-dependent predictor and functional status during five time points as dependent variables. These analyses were repeated for the six WHODAS domain scores separately.

The interaction term depression severity × time was added to the fully adjusted model to assess whether the course of functional limitations differed according to the severity level of depression.

Multi-collinearity of variables included in the final model was tested with correlation matrices. All correlations between variables were < 0.60, therefore none of the variables were excluded from the final model.

All *p* values were tested two-tailed and *p* values ≤ 0.05 were considered statistically significant. Data were analysed using Statistical Package of the Social Sciences (SPSS), version 22.

## Results

The mean age of the participants (*n* = 396) at baseline was 70.2 [standard deviation (SD) = 7.3] years and 64.6% were female. Baseline characteristics of the remitted and non-remitted participants, and the non-depressed comparison group are shown in Table 1. Group differences were found with respect to educational level, cognitive functioning, presence of multimorbidity, severity of depressive symptoms, total functional limitations and functional limitations on all six domains.

**Table 1:** Characteristics of the study population (*N* = 396)

Baseline characteristics	Non-depressed comparison group (*N* = 111)	Remission after 2 years (*N* = 147)	Non-remission after 2 years (*N* = 138)	p*	Post hoc analysis (*p* ≤ 0.05)^†^
Age, mean (SD)	69.3 (6.7)	70.4 (7.1)	70.8 (7.9)	0.237	
Female sex, %	62.1	65.2	66.0	0.805	
Education, mean (SD), years	12.5 (3.3)	10.7 (3.2)	(3.7)	< 0.001	1 > 2, 1 > 3
MMSE score, mean (SD)	28.5 (1.3)	27.8 (2.9)	27.7 (1.8)	0.014	1 < 2, 1 < 3
Multimorbidity, %	27.9	40.8	50.0	0.002	1 < 2, 1 < 3
IDS score, mean (SD)	7.0 (5.4)	25.1 (12.2)	(12.5)	< 0.001	1 < 2, 1 < 3, 2 < 3
IDS score at 2 years, mean (SD)	7.2 (5.5)	16.8 (9.6)	31.0 (11.9)	< 0.001	1 < 2, 1 < 3, 2 < 3
WHODAS 32 items (0–100), mean (SD)	5.7 (6.2)	20.8 (12.4)	(12.9)	< 0.001	1 < 2, 1 < 3, 2 < 3
WHODAS 32 items (0–100) at 2 years, mean (SD)	6.9 (7.1)	17.6 (11.3)	27.5 (14.7)	< 0.001	1 < 2, 1 < 3, 2 < 3
Domains, mean (SD)					
Understanding and communicating	6.2 (8.3)	24.4 (17.5)	32.7 (17.6)	< 0.001	1 < 2, 1 < 3, 2 < 3
Getting around	6.5 (12.4)	19.2 (20.2)	30.4 (23.1)	< 0.001	1 < 2, 1 < 3, 2 < 3
Self care	2.0 (6.6)	10.2 (12.2)	14.2 (14.8)	< 0.001	1 < 2, 1 < 3, 2 < 3
Getting along with people	7.2 (8.8)	21.8 (14.2)	24.4 (14.9)	< 0.001	1 < 2, 1 < 3, 2 < 3
Household activities	8.0 (13.1)	23.4 (20.6)	31.3 (19.9)	< 0.001	1 < 2, 1 < 3, 2 < 3
Participation in society	4.7 (7.2)	24.5 (15.1)	30.0 (15.5)	< 0.001	1 < 2, 1 < 3, 2 < 3
Domains at 2 years, mean (SD)					
Understanding and communicating	6.6 (8.4)	21.4 (20.6)	32.1 (18.5)	< 0.001	1 < 2, 1 < 3, 2 < 3
Getting around	9.1 (14.7)	22.0 (20.6)	32.0 (22.1)	< 0.001	1 < 2, 1 < 3, 2 < 3
Self care	1.7 (4.9)	9.1 (13.0)	14.8 (15.0)	< 0.001	1 < 2, 1 < 3, 2 < 3
Getting along with people	8.0 (8.4)	18.0 (12.9)	29.2 (15.1)	< 0.001	1 < 2, 1 < 3, 2 < 3
Household activities	10.8 (15.5)	22.0 (18.8)	32.9 (20.2)	< 0.001	1 < 2, 1 < 3, 2 < 3
Participation in society	5.7 (7.5)	14.7 (13.0)	28.8 (15.7)	< 0.001	1 < 2, 1 < 3, 2 < 3

*IDS* Inventory of depressive symptomatology, *MMSE* Mini–Mental State Examination, *WHODAS* WHO-disability assessment schedule

*Overall group differences. Comparison using ANOVA (continuous variables) and *χ*^2^ statistics (categorical variables)

^†^Groups in post hoc analysis noted as: 1 = non-depressed comparison group, 2 = patient in remission after 2 years, 3 = non-remitted patient after 2 years

### Remission

Linear mixed model analyses showed that, despite improvements in the first 6 months of follow-up, both the non-remitted group and the remitted group reported significantly worse functioning over the 2 years of follow-up, compared to the non-depressed comparison group (*β* = 20.51, SE = 1.23, *p* ≤ 0.001, and *β* = 11.80, SE = 1.19, *p* ≤ 0.001).

When the non-remitted group was compared to the group that was remitted after 2 years, we found that non-remission was associated with more severe functional limitations during these 2 years (*β* = 8.71, SE = 1.10, *p* ≤ 0.001).

**Fig. 1:**
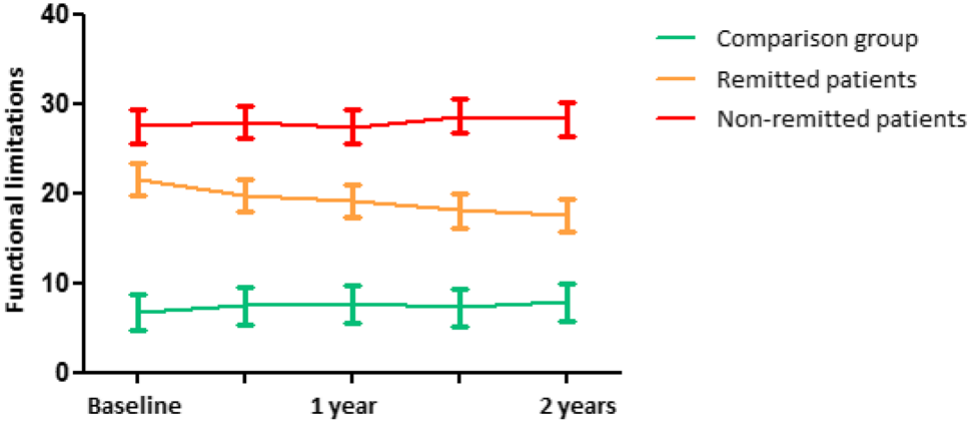
Course of functioning (estimated means) with 95% confidence intervals over 2 years for patients remitted from depression (*N* = 147), non-remitted patients (*N* = 138) and the non-depressed comparison group (*N* = 111)*^,†,‡^. *Based on linear mixed model analyses adjusted for age, sex, level of education, Mini–Mental State Examination score, multimorbidity and time. ^†^Interaction effect group × time: *p* ≤ 0.001 (remitted patients), *p* = 0.983 (non-remitted patients), comparison group = reference group. Remitted patients versus non-remitted patients: *p* < 0.001. ^‡^Higher scores reflect a higher level of disability. *IDS* Inventory of depressive symptomatology

Furthermore, by adding the interaction term group × time to the model, we found that the remitted group showed significant improvements in overall functioning over 2 years, although they did not reach the level of functioning of the comparison group (Fig. 1). Figure 1 shows that, the pace of the decline did not differ between the non-remitted group and the comparison group.

**Fig. 2:**
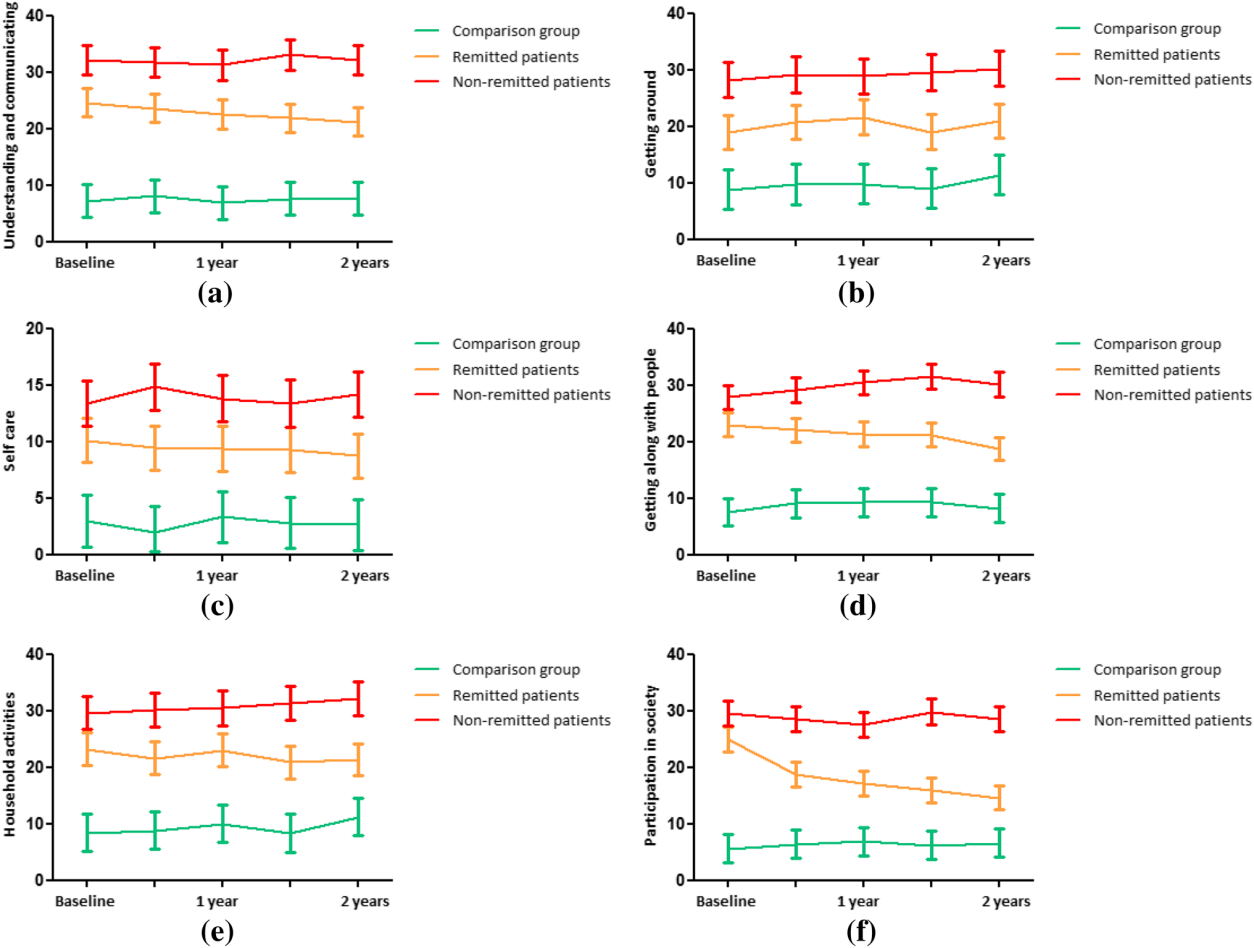
**a**–**f** Course of functioning (estimated means) with 95% confidence intervals over 2 years for patients remitted from depression (*N* = 147), non-remitted patients (*N* = 138) and the non-depressed comparison group (*N* = 111)*^,†,‡^. Based on linear mixed model analyses adjusted for age, sex, level of education, Mini–Mental State Examination score, multimorbidity and time. **a** Interaction effect group × time: *p* = 0.023 (remitted patients), *p* = 0.791 (non-remitted patients), comparison group = reference group. Remitted patients versus non-remitted patients: *p* = 0.007. **b** Interaction effect group × time: *p* = 0.589 (remitted patients), *p* = 0.978 (non-remitted patients), comparison group = reference group. Remitted patients versus non-remitted patients: *p* = 0.588. **c** Interaction effect group × time: *p* = 0.348 (remitted patients), *p* = 0.940 (non-remitted patients), comparison group = reference group. Remitted patients versus non-remitted patients: *p* = 0.283. **d** Interaction effect group × time: *p* = 0.001 (remitted patients), *p* = 0.121 (non-remitted patients), comparison group = reference group. Remitted patients versus non-remitted patients: *p* < 0.001. **e** Interaction effect group × time: *p* = 0.030 (remitted patients), *p* = 0.797 (non-remitted patients), comparison group = reference group. Remitted patients versus non-remitted patients: *p* = 0.010. **f** Interaction effect group × time: *p* < 0.001 (remitted patients), *p* = 0.489 (non-remitted patients), comparison group = reference group. Remitted patients versus non-remitted patients: *p* < 0.001. Higher scores reflect a higher level of disability. *IDS* Inventory of depressive symptomatology

The group × time interaction analyses were repeated for the six domains of functioning. Comparisons between all three groups were made. We found different courses of functioning for the following domains: understanding and communicating, getting along with people, household activities and participation in society (Fig. 2a–f). These differences were specifically found between the remitted group and the comparison group, and between the remitted group versus the non-remitted group. No course differences were found between the non-remitted group and the comparison group in any domain, implying similar courses of functioning over time between these two groups (Fig. 2a–f).

The domain that showed the largest difference between the remitted and the non-remitted group was participation in society (Fig. 2f).

### Severity of depression

The following analyses were carried out in the group that was depressed at baseline (*n* = 285). Table 2 shows that higher severity of depression was associated with more severe functional limitations during 2 years in the unadjusted and in the fully adjusted models. Severity of depression showed longitudinal associations with all six domains of functioning.

**Table 2:** Linear mixed model analysis of the longitudinal association between depressive symptoms (IDS Score) and functional limitations in the depressed subgroup

Functional limitations	Total score		Understanding and communicating		Getting around		Self care		Getting along with people		Household activities		Participation in society	
*N* = 285	*β* (SE)	*p*	*β* (SE)	*p*	*β* (SE)	*p*	*β* (SE)	*p*	*β* (SE)	*p*	*β* (SE)	*p*	*β* (SE)	*p*
Depressive symptoms														
Unadjusted	0.60 (0.02)	< 0.001	0.70 (0.03)	< 0.001	0.46 (0.04)	< 0.001	0.38 (0.03)	< 0.001	0.45 (0.03)	< 0.001	0.63 (0.04)	< 0.001	0.76 (0.03)	< 0.001
Adjusted*	0.59 (0.02)	< 0.001	0.70 (0.03)	< 0.001	0.46 (0.04)	< 0.001	0.37 (0.03)	< 0.001	0.46 (0.03)	< 0.001	0.62 (0.04)	< 0.001	0.75 (0.03)	< 0.001
IDS score × time	0.02 (0.01)	0.077	0.02 (0.02)	0.284	0.00 (0.02)	0.948	0.01 (0.01)	0.294	0.03 (0.01)	0.011	0.00 (0.02)	0.884	0.03 (0.01)	0.038

*IDS* Inventory of depressive symptomatology

*Adjusted for age, sex, level of education, Mini–Mental State Examination score, multimorbidity and time

When interaction term time × depression severity was added to the fully adjusted model, we found an almost statistical significant association between higher severity of depressive symptoms and an adverse course of functioning over time (*β* = 0.02, SE = 0.01, *p* = 0.077). When examining the individual domains, we found statistically significant associations for increasing severity of depression with an adverse course of the domains ‘getting along with people’ (*β* = 0.03, SE = 0.01, *p* = 0.011) and ‘participation in society’ (*β* = 0.03, SE = 0.01, *p* = 0.038) (Table 2).

## Discussion

Our results show that remission from depression is accompanied by improvements in functioning. Nonetheless, compared to a non-depressed comparison group, significant functional limitations remain, even when adjusted for presence of somatic diseases. This suggests that remission from depression not necessarily implies functional recovery. In the non-remitted group as well as the non-depressed comparison group we found a small, gradual increase of functional limitations over the 2-year follow-up as can be expected in a sample of older adults.

Incomplete recovery of functional limitations among participants who achieve remission may be explained by the presence of subthreshold symptoms of depression, inflating functional limitation scores [4, 12, 30, 31]. In older persons, relapse rates of depression are higher than in younger adults, and although direct comparisons are lacking, subthreshold depression is assumed to be more prevalent in older persons than in persons aged < 65 years [32–36]. Previous research has shown that subclinical depression is also associated with functional decline [37]. In line with this explanation is the dose–response effect that seems to be present in the depressed subgroup, since more severe depressive symptoms predict more functional decline. Residual depressive symptoms and related functional impairment not only worsen quality of life but also increase the risk for a new depressive episode [6].

Another explanation may be that participants with remitted depression had worse levels of functioning even before the onset of depression, compared to the non-depressed comparison group. This hypothesis is supported by findings from Ormel et al. [38] who found that prior to the onset of depression, functioning is already impaired. Another study found that although post-morbid levels of functioning were similar to pre-morbid levels of functioning, they differed from those of non-depressed persons [39], suggesting that the functional limitations we found in remitted participants may be part of a pre-existing vulnerability.

A third explanation may be that remission from depression in later life not necessarily leads to recovery of the consequences of a depressive episode. An example to illustrate this pertains to social contacts. If a depressed person neglects his/her social network during the depressive episode, remission from depression does not imply a restored social network. It will take time and effort to restore the network. The consequences of depression persist after remission and this simply conveys that a new treatment phase should be added to the treatment of depression in later life, with a focus on functional recovery. Since relapse and recurrence of depression in later life are common [35, 36], expanding treatment options is very welcome in old-age psychiatry.

The latter explanation is important for clinical practice, since this implies that treatment should focus on more than remission from depression. The domains of functioning provide more detailed information about which domains are affected by depression. We found that the domains that are more socially oriented (understanding and communicating, getting along with people and participation in society) and household activities developed differently over time for the non-remitted participants, the remitted participants and the comparison group. Higher recovery rates were found for the more socially oriented domains within the remitted group. This is not surprising, because depression directly affects social functioning [40].

Depression treatment should aim at complete clinical and functional recovery. A previous study in younger adults already showed that functional recovery takes longer in patients with recurrent mood disorders when compared to patients with a first or single episode [6]. For older adults, focusing on functional recovery is particularly important, because functional limitations may restrict older persons from living at home. Besides the financial benefits of living at home instead of a nursing home, maintaining independence is of great value for older persons [41]. It was already shown that nurse-led, home-based depression care reduces the risk of hospital admission, especially in patients with moderate to severe depression [42]. Combining the results from our study with the knowledge from previous research, guides us towards designing personalized, enduring care for persons suffering from depression in later life.

### Methodological considerations

To our knowledge, this is the first study that has examined the association between depression in later life and functional decline prospectively. We included a sample of older persons with a DSM-IV confirmed depression diagnosis, contrary to previous studies that used questionnaires as an indicator of depression status. In addition to the formal depression diagnosis, this study used a well-validated measure of severity of depressive symptoms (IDS), enabling us to perform in-depth analyses of the longitudinal associations between severity of depressive symptoms and functional limitations. Data were collected from different treatment settings, covering the whole spectrum of depressive disorders. This enhances the generalizability of the results.

The limitations of the study include drop-out during the follow-up period of participants that were more severely depressed at baseline and had more functional limitations than the participants that were included in the analytical sample, suggesting that the most severely impaired persons were not followed up. This may have biased our results towards and underestimation of the association that was found between depressive symptoms and functional decline. Furthermore, our study did not assess pre-morbid levels of functioning. Therefore, we cannot take a possible pre-existing vulnerability into account. Lastly, NESDO cohort is a naturalistic cohort of depressed older persons, therefore, including participants with early and late-onset of depression. The role of age of onset in the association between depression and functional limitations is unknown, and the analyses were not corrected for age of onset. The literature does show that early onset of depression is associated with an unfavourable course of depression [43].

## Conclusion

Our study showed that there is a considerable gap between symptomatic and functional recovery in patients suffering from depression in later life. The negative impact of depression on functional limitations points to the importance of incorporating collaborative, multi-facetted, nurse-led interventions in the treatment of depression in later life [44, 45]. Further understanding of the mediating mechanisms underlying the association between depression and functional limitations may guide the development of these interventions.
